# End-to-end design of metasurface-based complex-amplitude holograms by physics-driven deep neural networks

**DOI:** 10.1515/nanoph-2022-0111

**Published:** 2022-05-11

**Authors:** Wei Wei, Ping Tang, Jingzhu Shao, Jiang Zhu, Xiangyu Zhao, Chongzhao Wu

**Affiliations:** Center for Biophotonics, Institute of Medical Robotics, Shanghai Jiao Tong University, Shanghai, China; School of Biomedical Engineering, Shanghai Jiao Tong University, Shanghai, China

**Keywords:** complex-amplitude hologram, inverse design, metasurface, neural network

## Abstract

Holograms which reconstruct the transverse profile of light with complex-amplitude information have demonstrated more excellent performances with an improved signal-to-noise ratio compared with those containing amplitude-only and phase-only information. Metasurfaces have been widely utilized for complex-amplitude holograms owing to its capability of arbitrary light modulation at a subwavelength scale which conventional holographic devices cannot achieve. However, existing methods for metasurface-based complex-amplitude hologram design employ single back-diffraction propagation and rely on the artificial blocks which are able to independently and completely control both amplitude and phase. Here, we propose an unsupervised physics-driven deep neural network for the design of metasurface-based complex-amplitude holograms using artificial blocks with incomplete light modulation. This method integrates a neural network module with a forward physical propagation module and directly maps geometric parameters of the blocks to holographic images for end-to-end design. The perfect reconstruction of holographic images verified by numerical simulations has demonstrated that compared with the complete blocks, an efficient utilization, association and cooperation of the limited artificial blocks can achieve reconstruction performance as well. Furthermore, more restricted controls of the incident light are adopted for robustness test. The proposed method offers a real-time and robust way towards large-scale ideal holographic displays with subwavelength resolution.

## 1 Introduction

Conventional optical holography records the fringe pattern formed by the scattered field of the object and the coherent reference field on a photosensitive film, and demands the complicated step of developing in the fabrication process. Without requirements for real objects or coherent light sources, computer-generated holography (CGH) has been serving as an effective way to digitally generate the hologram by inverse design algorithm on computers [1]. In CGH, the field distribution at the hologram plane is calculated numerically from a given virtual object and then coded into a spatial light modulator (SLM) to display the target object over a certain distance. Owing to its excellent capacity of shaping wavefront and synthesizing beams to desired intensity distributions, CGH is extensively used in 3D holographic display [2], optical encryption [3] and optical data storage [4]. However, in terms of phase accumulation over propagation distances, wavefront manipulation devices widely employed in CGH, such as SLMs, have to possess much more bulky geometry size compared to the working wavelength. Furthermore, the resolution of holographic images is limited by the pixel size of SLMs, which is usually in the range of microns.

Based on the modulation type, CGH can be classified into phase-only holograms, amplitude-only holograms, and complex-amplitude holograms. Phase-only and amplitude-only holograms are extensively used in SLM-based holography because current SLMs cannot achieve simultaneous modulation of both phase and amplitude, and these holograms lack either phase or amplitude information, suffering from the speckle noises on the reconstructive images compared with complex-amplitude holograms.

Metasurface-based holograms (MHs) has been emerging technologies to overcome the challenges above. Metasurfaces are flat optical elements which are composed of periodic artificial blocks at subwavelength scales and have been used to manipulate electromagnetic field with low absorption loss and a reduced complexity of fabrication [5, 6]. Each artificial block of metasurface, named unit cell, can achieve local phase and amplitude control of light, leading to flexible designs for metasurfaces with high degree of freedom. Owing to the ability of arbitrary manipulation of light, metasurfaces have demonstrated various applications in augmented reality [7], metalens [8], computational imaging [9], etc. Especially in CGH, ultra-compact MHs have been explored with high diffraction efficiency [10], [11], [12], [13], [14], [15], multicolor display [16], [17], [18], [19] and polarization-multiplexing [20], [21], [22], [23], [24]. With precise design of the geometric parameters of subwavelength unit cells, the wavefront controlled by metasurfaces would display a high-quality target holographic image with subwavelength resolution.

**Figure 1:**
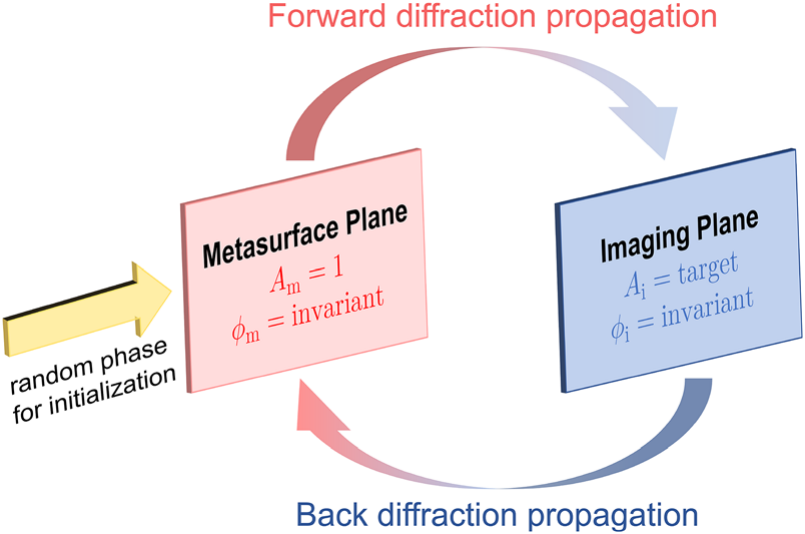
The flow chart of iterative Fourier-transform algorithms (IFTA) method for phase retrieval of a metasurface plane. Electric field propagates to the imaging plane from the metasurface plane with a random phase and unit amplitude as initialization, and the amplitude *A*_i_ is replaced by the target pattern while the phase *ϕ*_i_ keeps invariant. The field then propagates back to the metasurface plane and the amplitude *A*_m_ is replaced by unit. The process above runs iteratively until *A*_i_ is consistent with the target pattern.

Unfortunately, solving the geometric parameters of unit-cell array for a given target image is an ill-posed inverse problem because the solution is not unique and sensitive to the initial conditions. Iterative Fourier-transform algorithms (IFTA), such as Gerchberg–Saxton (GS) algorithm [25] and Yang–Gu algorithm [26], play a dominant role in existing hologram designs. For a given phase retrieval problem shown in Figure 1, the phase mask of metasurface *ϕ*_m_ can be generated from the iterative forward and back diffraction propagations between the electric fields in the metasurface plane *E*_m_ and imaging plane *E*_i_ starting with a random phase value. The IFTA method is simple and straightforward but needs extensive computational time when image pixels reach the orders of millions. Meanwhile, it should be pointed out that GS algorithm can only generate a phase-only hologram due to the amplitude normalization in the metasurface plane, leading to the requirement for unit cells of full phase modulation with constant transmission. Thus GS algorithm needs to be modified for designing holograms with other specific functions [27]. In the field of complex-amplitude holograms, the phase and amplitude in the metasurface plane are calculated through single back diffraction propagation without amplitude normalization, which requires complete and simultaneous control of the phase and amplitude. Both phase-only and complex-amplitude MHs require the special capacity of light modulation, resulting in the difficulty seeking out a cluster of unit cells to meet the demand above, particularly in complex-amplitude MHs [16, 28], [29], [30]. Hence, a new algorithm is highly desired for MHs design with limited freedom of light modulation.

Recently, machine learning-based methods have gained more and more attention for the powerful abilities to characterize the intrinsic relationships among large amounts of samples, and have been widely used in analysis, design and optimization of various photonic structures [31, 32]. With unprecedented breakthroughs of deep neural network (DNN) during the last a few years, the methods for inverse design of photonics devices have got rid of human-based trial and error and embraced the data-driven machine learning technology to explore the optimal design parameters. DNN is structured with multilayer nonlinear operators and learns from massive labeled dataset to “fit” the inverse model. Take into account that creating a training dataset of MHs is impractical either from experiments or numerical simulations according to enormous degrees of freedom over MH designs [33], the unsupervised neural network exactly meets the demands because a preparation for labeled dataset is not required. Recent advances have demonstrated unsupervised neural network enabled designs for CGH. An unsupervised neural network model, named DeepCGH, has proposed in [34] for SLM-based holography. An unsupervised conditional generative adversarial network is adopted in [35] for MHs design, however, the states of the unit cell only contain “0” and “1”, corresponding to phase shift of 0 and π, with no capacity of continuous phase control. What’s more, the unit cells lack the ability to control amplitude so that the network in [35] could generate phase-only metasurface holograms only.

**Figure 2:**
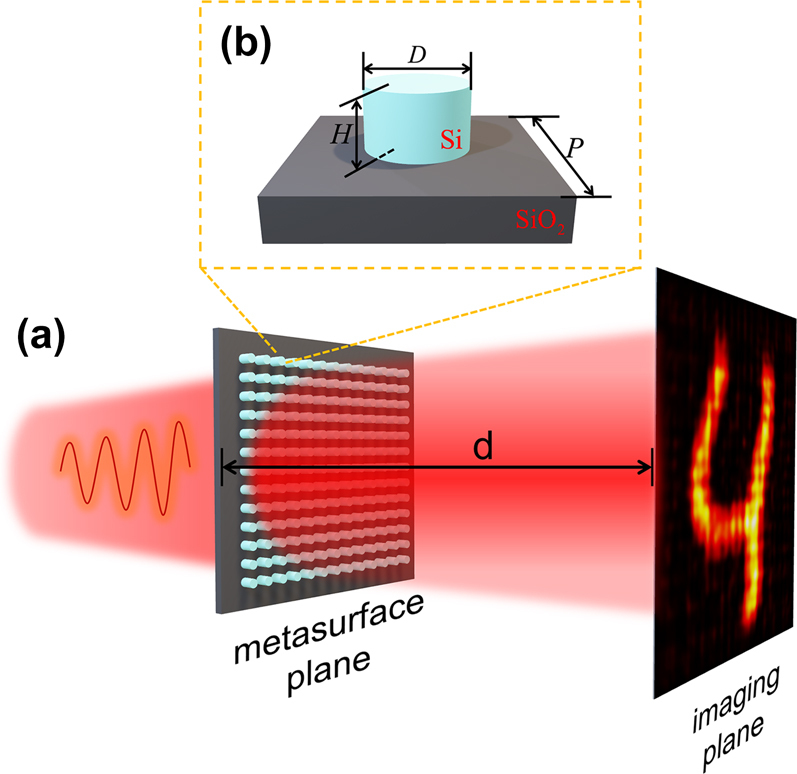
A schematic view of the metasurface-based hologram. The coherent plane wave passes through the designed metasurface, and a target holographic image is displayed at the imaging plane after a diffraction propagation distance *d*. Inset shows the diameter (*D*) and height (*H*) of the Si-cylinder cell, which serve as the designed parameters in the section of Methods, and *P* denotes the period of nanocylinder array.

In this work, we propose an unsupervised physics-driven deep neural network for the design of metasurface-based complex-amplitude holograms using the unit cells with incomplete modulation on both phase and amplitude. The method serves as a generative adversarial model while neural network module is the generator and physical propagation module is the discriminator. Silicon nanocylinder with propagation-phase modulation is utilized as the unit cell. The diameter and height of the cylinder are selected as design parameters, as shown in Figure 2. When appropriately modifying geometric parameters of cylinders, the effective refractive index of each cylinder waveguide can be manipulated individually to shape the wavefront and manipulate the intensity. To the best of our knowledge, it is the first time to demonstrate an end-to-end design of complex-amplitude metasurface-based holograms from geometric parameters of unit array directly to the holographic image without requirements for complete light modulation. Taking advantages of such one-step process with ultrafast design speed (less than 1 s once the training procedure is completed), the method in this work will be beneficial to the real-time and large-scale holographic displays with subwavelength resolution.

In the section of Methods, the specific architectures of neural network and physical mechanism of light propagation will be introduced. In the section of Results, the results of designed metasurfaces from the neural networks and the comparisons with existing algorithms will be demonstrated. To evaluate the robustness of our algorithm, light modulations with different degrees of comfinement are tested. Detailed numerical simulations are carried out for validation as well.

## 2 Methods

**Figure 3:**
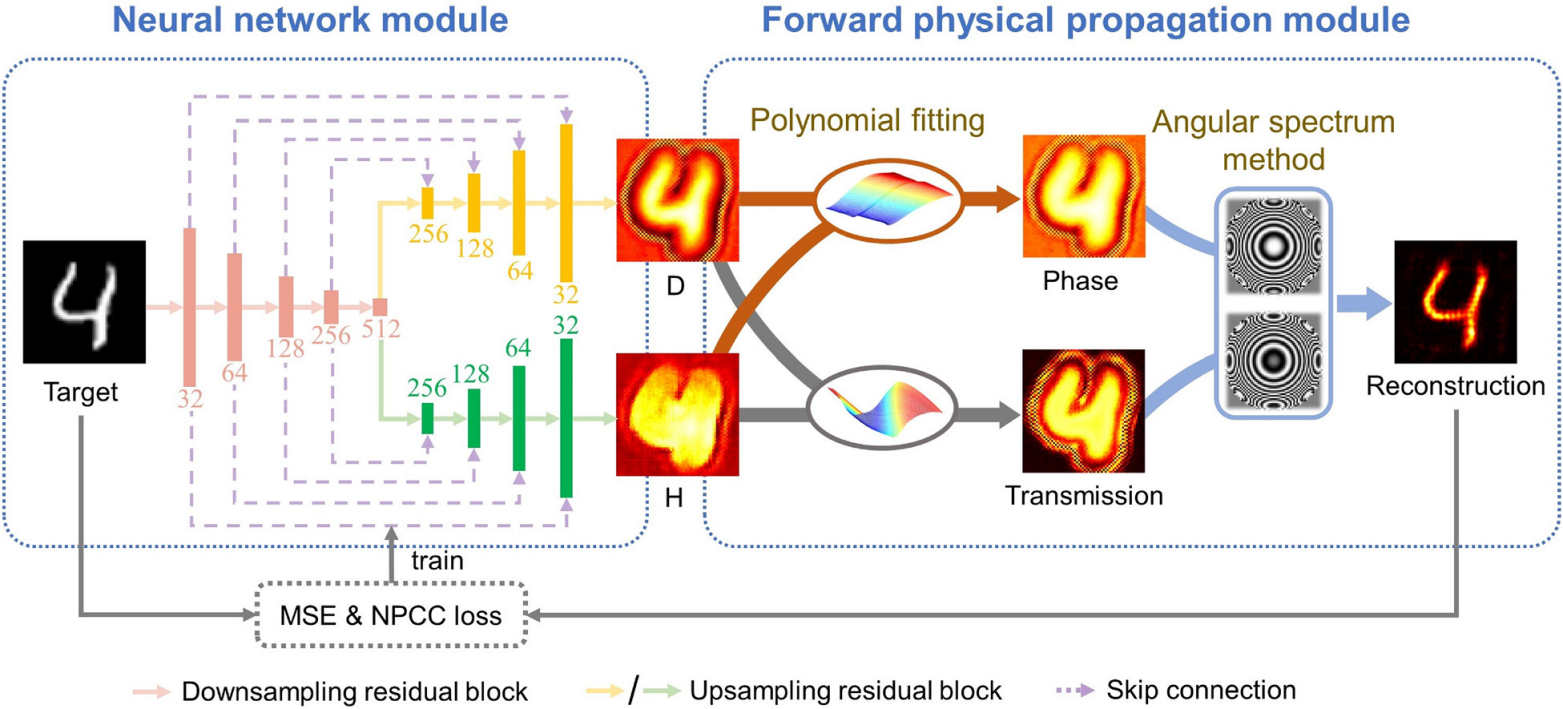
Architecture of the network with cooperation of two modules, i.e., neural network module and forward physical propagation module. The output image of forward physical propagation module is reconstructed to the same pattern as the target image to obtain the diameter map and the height map of nanocylinder array.

An architecture of the entire framework is illustrated in Figure 3. The deep neural network module operates as a generator to output height and diameter maps of nanocylinder array corresponding to the target holographic image. The target image is reconstructed in imaging plane by the means of diffraction propagation after sending height and diameter maps into the forward physical propagation module, which serves as the discriminator. Loss between reconstructive images and target images is calculated to drive the training process.

### 2.1 Neural network module

We adopt a Y-shape deep neural network, called Y-Net, to generate the diameter map and the height map simultaneously. The Y-Net consists of five down-sampling residual blocks [36] as the encoder to capture the discriminative features of target electric field (E-field) map, and two paths, each with five up-sampling residual blocks, as the decoders for precise retrieval of diameter map and height map, respectively, as shown by the left dashed box in Figure 3, in which numbers denote the channels of feature maps and the sampling rate is set as 2-fold for each residual block. Moreover, skip connections are employed between each of up- and down-sampling residual blocks to skip multi-scale spatial information from encoder to decoder and enhance the high-resolution reconstruction. The activation function in output layer is Sigmoid, while others adopt ReLU [37] for activation. Besides, the learning rate is set as 10^−4^, and batch size as 128.

The combination of mean square error (MSE) and negative Pearson correlation coefficient (NPCC) between target E-field map and reconstructive E-field map is introduced as loss function to drive the back-propagation of Y-Net, and is defined as:

(1) $${\text{Loss}}_{\text{total}}=\tau \cdot {\text{Loss}}_{\text{MSE}}+{\text{Loss}}_{\text{NPCC}}$$

(2) $${\text{Loss}}_{\text{NPCC}}=\frac{\sum_{i}\left(I_{\text{tar},\text{i}}-{\bar{I}}_{\text{tar}}\right)\left(I_{\text{re},\text{i}}-{\bar{I}}_{\text{re}}\right)}{\sqrt{\sum_{i}{\left(I_{\text{tar},\text{i}}-{\bar{I}}_{\text{tar}}\right)}^{2}\sum_{i}{\left(I_{\text{re},\text{i}}-{\bar{I}}_{\text{re}}\right)}^{2}}}$$

(3) $${\text{Loss}}_{\text{MSE}}=\frac{1}{N}\sum_{i}^{N}{\left\|I_{\text{tar},\text{i}}-I_{\text{re},\text{i}}\right\|}^{2}$$

where *I*_tar_ and *I*_re_ represent the E-field intensity of target images and reconstructive images, while ${\bar{I}}_{\text{tar}}$ and ${\bar{I}}_{\text{re}}$ donate the average value of itself, respectively. Loss_MSE_ acts as data constraint to penalize the average error between each pair of pixels in target and reconstructive images of E-field, while Loss_NPCC_ focuses on the global similarity between different intensity distributions (Detailed comparisons can be found in Figure S1 of Supplementary Materials). *τ* is the weight to balance Loss_MSE_ and Loss_NPCC_ and ranges from 0.1 to 1 for a robust reconstruction. In this work, *τ* is uniformly set as 1. Moreover, Euler’s equation is employed to obtain the gradients of the complex values of E-field that are involved in the back-propagation process of our neural network by splitting the complex values into individual real and imaginary parts and calculating the gradient of these parts, respectively [38].

### 2.2 Forward physical propagation module

The diameter maps and height maps output from Y-Net are then sent to the forward physical propagation module for reconstruction of target E-field patterns. The forward physical propagation model here drives the E-field propagation process from metasurface holograms to the target holographic patterns in imaging plane.

The forward physical propagation module consists of a polynomial fitting step to formulate the phase and transmission modulation as the function of height and diameter, as shown in Figure 3 by the orange red and deep gray thick curves, respectively, and a step of angular spectrum method to transfer E-field from metasurface plane to imaging plane, as shown by the light blue thick curves.

**Figure 4:**
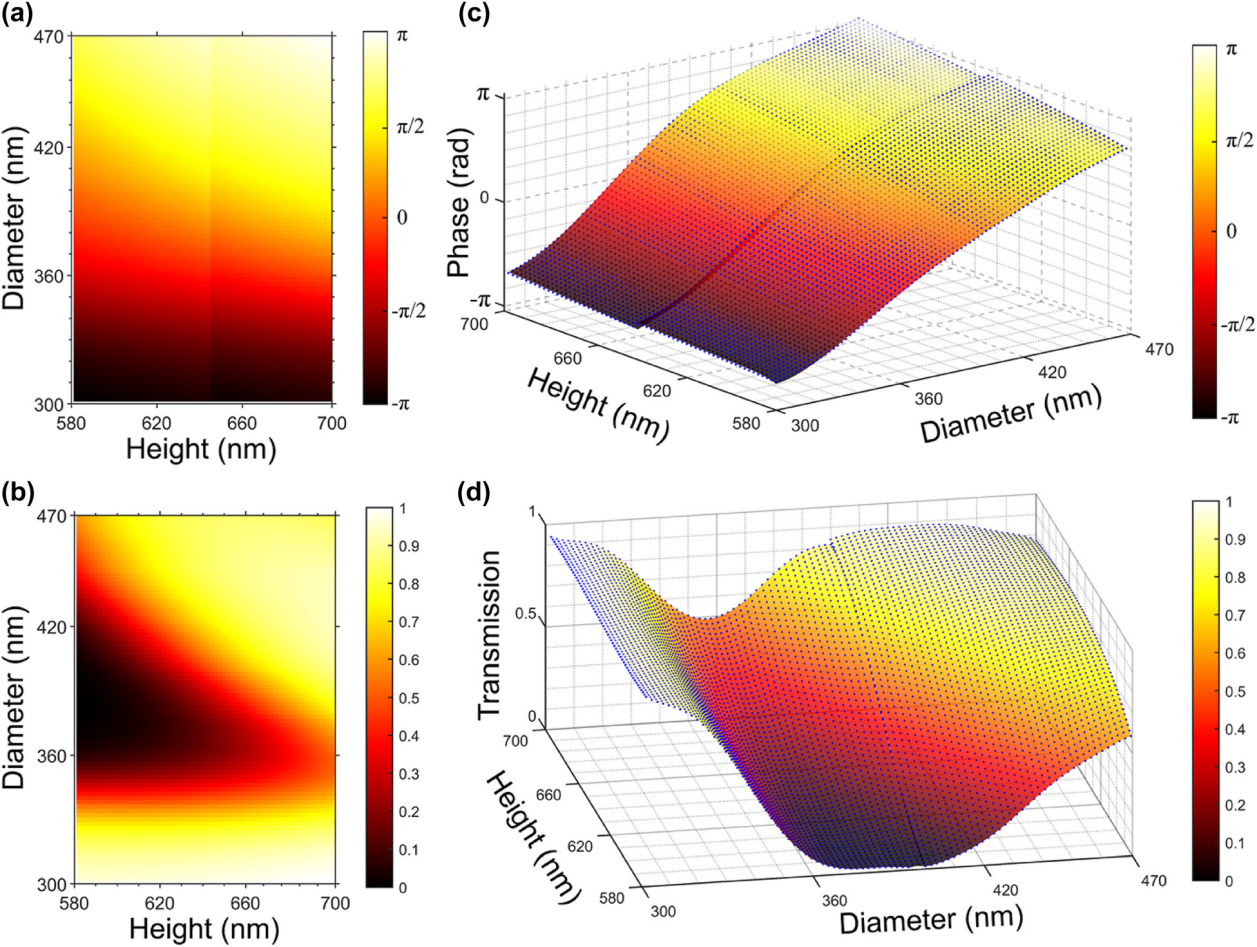
Phase shift and transmission response of Si nanocylinders with various diameters and heights at the wavelength of 1.5 µm. (a) and (b) The simulation results from FDTD for phase shift and transmission, respectively. The diameter of nanocylinders ranges from 300 to 470 nm, while the height ranges from 580 to 700 nm, with a 2-nm interval for both diameter and height. (c) and (d) The fitting results by piecewise polynomial fitting method. The blue dots represent the exact values from FDTD.

Firstly, phase and transmission response relationships of metasurfaces with various geometric parameters are stablished through numerical simulations. As shown in Figure 2, the metasurface employed here consists of 64 × 64 nanocylinder array with a period (*P*) of 750 nm. The diameter and height of each nanocylinder, together with their locations, make up the spatial distribution maps, i.e., diameter map and height map, respectively. Numerical simulations are calculated using finite difference time domain (FDTD) method (Lumerical FDTD Solutions) to obtain the phase shift and amplitude attenuation (transmission) data by sweeping geometric parameters. Periodic boundary conditions are set among the in-plane directions and perfect matched layer (PML) is applied along the *z*-axes. A *x*-polarized plane-wave source with the wavelength of 1.5 µm is normally incident on the substrate and monitored 3-µm above the metasurface to obtain phase and transmission responses. Figure 4(a) and (b) show the simulation results of phase and transmission as a function of nanocylinder diameter and height. The diameter of nanocylinders ranges from 300 to 470 nm, while the height ranges from 580 to 700 nm, with a 2-nm interval for both. The limited ranges of diameter and height restrict the complete modulation to phase, amplitude and arbitrary combination of both, causing the unsuitability when adopting the existing algorithms of holographic design. Considering the back-propagation process, the phase and transmission responses to diameter and height are discrete and non-differentiable. Therefore, piecewise polynomial fitting method are leveraged to establish the continuous mappings, as illustrated in Figure 4(c) and (d), in which phase and transmission are formulated as a specific polynomial function of both diameter and height, respectively. The fitting errors in terms of MSE are 0.0112 and 0.0034 for phase and transmission, respectively.

When a light beam passes through the aperture at wavelength scale, instead of simple projection in the manner of geometrical optics, the scattered field will interfere reciprocally and complies with scalar diffraction theory, including Fresnel diffraction, Fraunhofer diffraction and angular spectrum propagation. The Fresnel diffraction and Fraunhofer diffraction employ paraxial approximation on the basis of angular spectrum propagation. Hence the angular spectrum method is more accurate for holograms with wide field of view and is adopted as the physical propagation algorithm in this paper. The complex E-field distribution in the phase mask plane can be resolved to plane wave fields with various spatial frequency components through two-dimensional Fourier transform, given by

(4) $$A_{\text{m}}\left(\frac{\alpha }{\lambda },\frac{\beta }{\lambda }\right)=\iint_{-\infty }^{+\infty }E_{\text{m}}\left(x,y\right)\text{exp}\left[-ik\left(\alpha x+\beta y\right)\right]\text{d}x\text{d}y$$

where *A*_m_ (*α*/*λ*, *β*/*λ*) represents the angular spectrum of plane wave field exp[−*ik*(*αx* + *βy*)] with *x*- and *y*-direction spatial frequencies *f*_*x*_ = *α*/*λ*, *f*_*y*_ = *β*/*λ*. The transfer function of angular spectrum can be solved from the Helmholtz equation to determine the angular spectrum in the imaging plane at a distance of *d*, that is,

(5) $$A_{\text{i}}\left(\frac{\alpha }{\lambda },\frac{\beta }{\lambda }\right)=A_{\text{m}}\left(\frac{\alpha }{\lambda },\frac{\beta }{\lambda }\right)\text{exp}\left(ikd\sqrt{1-\alpha^{2}-\beta^{2}}\right)$$

and then the E-field distribution *E*_i_ (*x*, *y*) in the imaging plane is derived through inverse Fourier transform,

(6) $$E_{\text{i}}\left(x,y\right)={ℱ}^{-1}\left\{A_{\text{i}}\left(\frac{\alpha }{\lambda },\frac{\beta }{\lambda }\right)\right\}$$

where ℱ^−1^{·} means the inverse Fourier transform.

The holographic image in this work consists of 64 × 64 pixels with pixel size of 750 nm, the same as that of the unit cell. To satisfy the Nyquist sampling condition for both amplitude and phase of E-fields in the two planes, the distance between two planes is set as 24 µm.

## 3 Results

### 3.1 Metasurface hologram design

**Figure 5:**
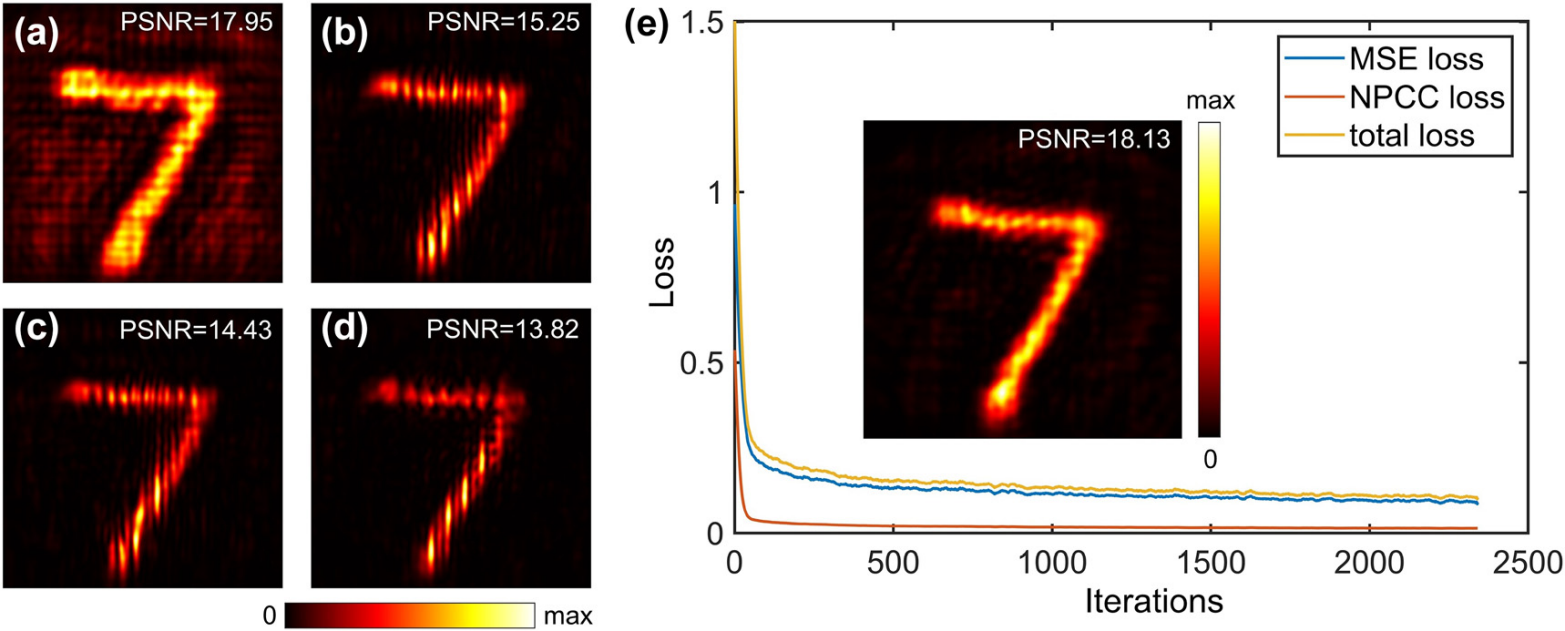
Intensity distribution of reconstructive images when the intensity of target images is normalized to different values. (a)–(d) FDTD results of normalization to 1, 5, 7 and 9, respectively. (e) Loss curve of the best reconstructive performance when the intensity of target images is normalized to 3 (Inset). The total loss decreases to 0.095 after ∼2000 iterations.

Modified National Institute of Standards and Technology (MNIST) datasets [39] of handwritten numbers are chosen as the target holographic images. Training dataset composed of 50,000 samples is used to train the proposed neural network model with a training batch size of 128 and a learning rate of 10^−4^. More details about the influence of parameters in neural network on the performance of proposed model can be found in Supplementary Materials Part I. Training the model is an unsupervised process with various target electric fields. The electric-field intensity value of reconstructive images is unknown before training, so the target electric field is normalized to various values to test the best performance. The performance of different intensity values is compared and validated by FDTD method in Figure 5. Peak signal-to-noise ratio (PSNR) of each normalization is calculated to evaluate the reconstruction performance. The best reconstruction occurs when the intensity is normalized to 3 and its loss curve during training stage decreases rapidly within dozens of epochs, indicating the reliability of the model, as illustrated in Figure 5(e). The proposed model is implemented in MATLAB R2020a equipped with a Deep Learning Toolbox and performed on a workstation with an Intel Xeon Silver 4210 (2.40 GHz, 11 cores), a GeForce RTX 2080 Ti and 30 GB RAM. Within the software and hardware support above, the generator, i.e., the proposed Y-Net model, takes about 2 h to converge and become stable, with the training loss decreasing from about 1.5 to less than 0.1, as shown by Figure 5(e). And once training procedure is completed, time for designing metasurface geometry, i.e., diameter and height maps, is within a second, which suggests that the proposed method is able to find potential applications in real-time holographic display.

**Figure 6:**
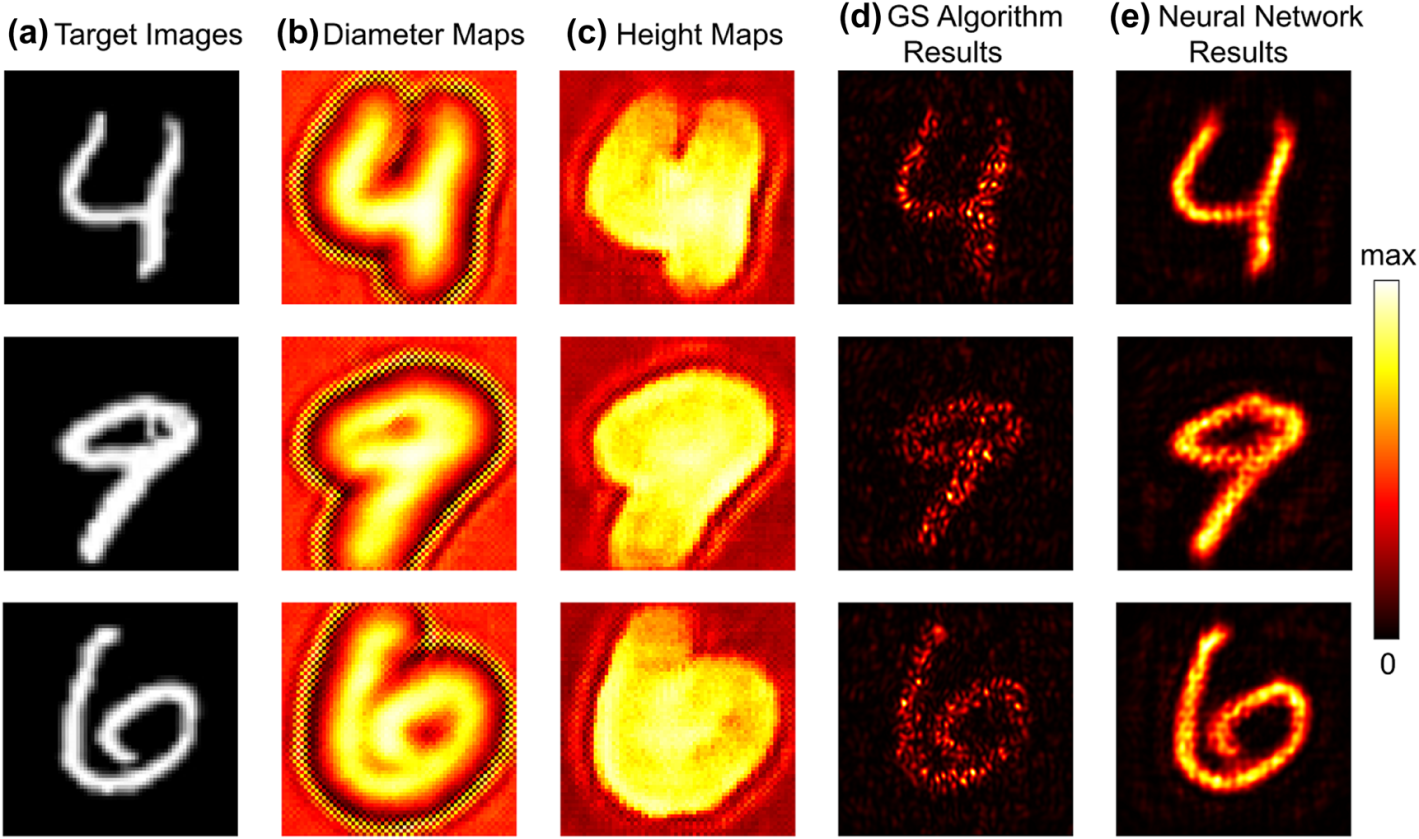
Testing results of the proposed physics-driven neural network compared with GS algorithm. (a) The target images. (b) and (c) The inverse design for diameter maps and height maps of metasurface-based holograms by the proposed neural network, respectively. (d) FDTD results of the reconstructive images from GS algorithm and (e) the proposed neural network, respectively, at a given distance of 24 µm.

Figure 6(b) and (c) illustrate the diameter maps and height maps generated by our deep neural network according to the target MNIST images in Figure 6(a). The holographic images from the well-designed MHs are calculated through forward physical propagation module. Furthermore, we utilize FDTD method to simulate the whole metasurfaces for validation of our model. PML is applied for all boundaries and the power monitor is placed at 24 µm above the metasurface to record the electric field shown in Figure 6(e). Reconstructive holographic images by both forward physical propagation module and FDTD method show a high similarity compared with the target images. The background is clean, and the energy distribution of electric field is confined in the target region with a sharp outline, which implies a high signal-to-noise ratio of the holographic image.

The performance of our proposed method is then compared with that of existing GS algorithm both intuitively and quantitively. It is worth noting that additional steps must be attached to generate maps of diameter and height following the GS algorithm, while our method is able to achieve an end-to-end inverse design. Figure 6(d) illustrates the FDTD results from GS algorithm. It can be found from Figure 6(d) and (e) that the neural network demonstrates a better performance intuitively, while the pattern generated from GS algorithm suffers from serious discontinuities. To measure the reconstruction quality quantitatively, PSNR and structural similarity (SSIM) are calculated for holographic images generated from the neural network method and GS algorithm, respectively. GS algorithm gets 12.7682 for PSNR and 0.1869 for SSIM, while the neural network gets 18.1988 for PSNR and 0.4233 for SSIM, suggesting a better performance with higher signal-to-noise ratio of the neural network. Due to the random phase initialization, the patterns of phase maps, diameter maps as well as height maps from GS algorithm show no correlation to their target images. Moreover, as shown by Figure 4(b), the transmission response varies with different diameter and heights of unit cells, causing that their amplitude responses could not achieve the maximum, which explains the poor performance of GS algorithm in this case.

To quantitively compare the reconstruction efficiency of the two methods, the average value of absolute E-field intensity |*E*|^2^ over the target region and the entire region of holographic image plane, named ${\bar{I}}_{\text{target}}$ and ${\bar{I}}_{\text{entire}}$ , respectively, is calculated. GS algorithm gets 0.4522 V^2^/m^2^ for ${\bar{I}}_{\text{entire}}$ , while the neural network method gets 0.5290 V^2^/m^2^. For ${\bar{I}}_{\text{target}}$ , GS algorithm gets 0.5923 V^2^/m^2^, while the neural network method gets 1.5309 V^2^/m^2^, which suggests that the proposed method achieves a much higher absolute intensity in the target region. We define

(7) $$R=\frac{\sum_{\text{target}}{\left|E\right|}^{2}}{\sum_{\text{entire}}{\left|E\right|}^{2}}\times 100\text{\%}$$

as the ratio of total electric-field intensity in the target imaging plane to that in the entire region to measure the efficiency of holographic reconstruction. By the means of neural network, 86.72% of electric-field energy in imaging plane is gathered to reconstruct the holographic image, compared with 39.41% by GS algorithm, demonstrating that the proposed method achieves much higher efficiency of reconstruction as well.

Furthermore, we have demonstrated the end-to-end inverse design of larger-scale metasurface holograms with array dimensions of 128 × 128, 256 × 256, 512 × 512, 1024 × 1024, respectively, using the proposed neural network method (more details in Supplementary Materials part II). The excellent reconstructive results (shown in Figure S3) imply that the proposed method is very promising for large-scale ideal holographic displays with subwavelength resolution.

### 3.2 Model robustness

The model robustness describes how restricted capacity of light modulation the model can withstand. As shown by Figures 4 and 6, the parameter range (300–470 nm for diameter & 580–700 nm for height) of the nanocylinders possesses incomplete phase and transmission control but achieves vivid reconstructive images.

**Figure 7:**
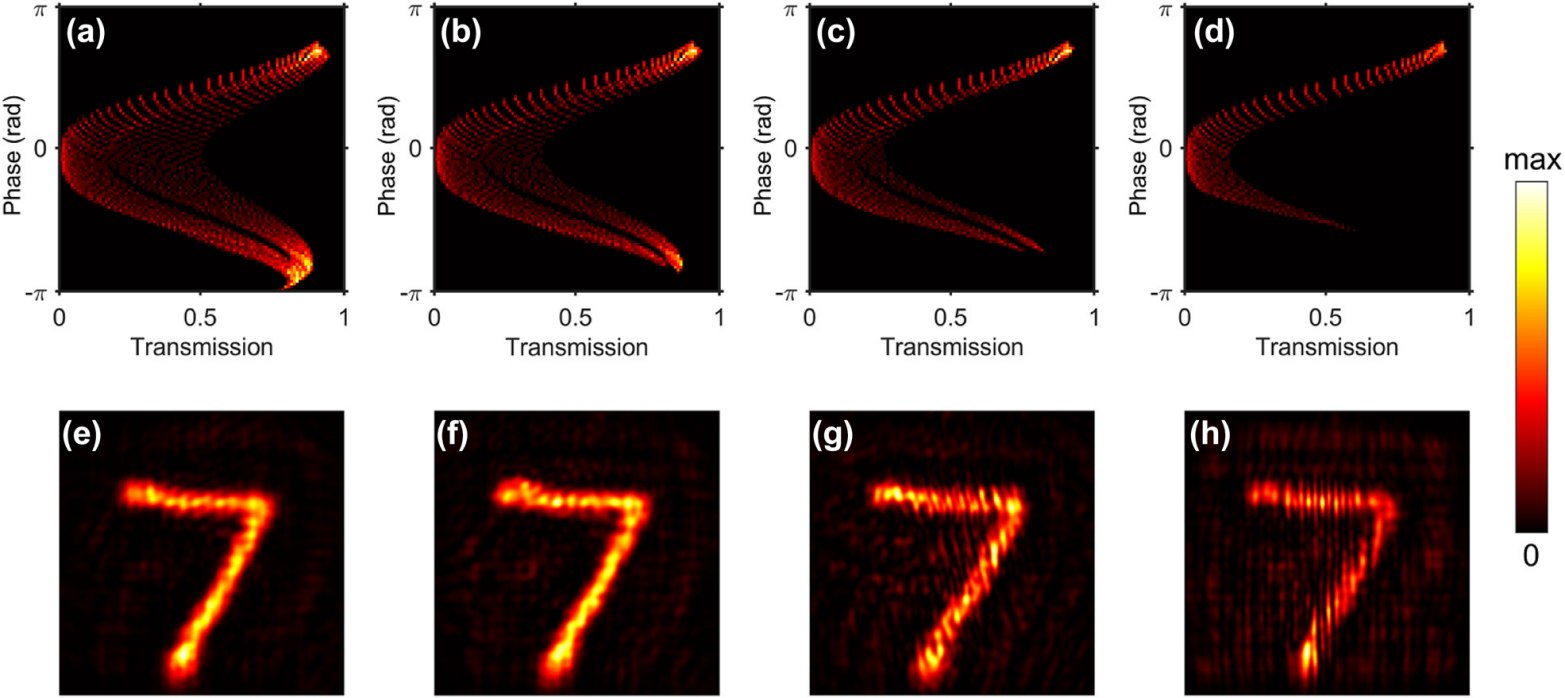
Capacity maps for phase and transmission modulation with varying ranges of the design parameters. The regions that lack the control of light is shown in black and get enlarged when the parameter ranges become narrower. (a) 300–470 nm for diameter & 580–700 nm for height that have been adopted in the section of Methods. (b) 300–450 nm for diameter & 580–680 nm for height. (c) 300–430 nm for diameter & 580–660 nm for height. (d) 300–410 nm for diameter & 580–640 nm for height. (e)–(h) Reconstructive images corresponding to (a)–(d).

To evaluate robustness of the model, tapered parameter ranges are adopted to generate light modulations with different degrees of confinement which are measured by the Phase-Transmission map here. The covering area of Phase-Transmission maps indicates the modulation capacity of different parameter ranges and the colormap shows the amount of nanocylinders that achieve the same modulation for both phase and transmission. Figure 7(a)–(d) shows the capacities for light modulation of the four tapered parameter ranges, covering 22.63%, 17.80%, 12.73% and 7.52% of the total, respectively.

The four phase and transmission responses of the parameter ranges above are utilized to train the physics-driven neural network, respectively. FDTD results are also calculated for validation. The metasurface hologram with narrower parameter range displays weaker reconstructive images, as shown in Figure 7(e)–(h). And even the narrowest tested parameter range presents an outstanding performance, indicating strong robustness of the algorithm.

## 4 Conclusions

In summary, a novel algorithm is demonstrated for the design of metasurface-based complex-amplitude holograms with the unit cells that are able to incompletely modulate phase and amplitude. The algorithm consists of the neural network module as the generator and the forward physical propagation module that drives the network unsupervisedly as the discriminator. FDTD method has been utilized to validate the reconstruction results of holographic images generated from the proposed physics-driven neural networks. Existing methods are inappropriate for the incomplete light modulation, suffering a poor performance as discussed in the section of Methods, and incapable of end-to-end design for unit cells. The scalability of the proposed method has been demonstrated by end-to-end inverse design of large-scale metasurface holograms with array dimensions up to 1024 × 1024. Tapered parameter ranges are employed to generate more restricted capacities of light modulation, indicating the strong robustness of the algorithm. This work demonstrated that complete modulation of light is not indispensable for complex-amplitude holograms. Instead of seeking a cluster of unit cells which meet demands in existing methods, the efficient utilization and association of limited unit cells releases more degrees of freedom for hologram design and is proved here to be an alternative and effective approach to realizing complex-amplitude holographic displays. More generally, the proposed method can be treated as a general design framework, including the neural network module and the forward physical propagation module, to obtain an array of design parameters corresponding to different target images. Working wavelength and the selected design parameters are regarded as the inputs of this general framework. Hence, the method still works with tunable working wavelength as inputs and has the potential to design active metasurfaces by choosing design parameters which can be modified dynamically. This work will also find broad applications in subwavelength real-time holography and inverse design of photonic devices.

## Supplementary Material

Supplementary Material
