# Supplementary material for: End-to-end design of metasurface-based complex-amplitude holograms by physics-driven deep neural networks
Source: Nanophotonics. 2022 May 11;11(12):2921–9. doi: 10.1515/nanoph-2022-0111 (PMC11501633; doi:10.1515/nanoph-2022-0111)
Supplement: Supplementary file 1 — Supplementary Material [file j_nanoph-2022-0111_suppl.docx]

*Nanophotonics*

**Supplementary Materials for**

End-to-end Design of Metasurface-based Complex-amplitude Holograms by Physics-driven Deep Neural Networks

Wei Wei^‡1,2^, Ping Tang^‡1,2^, Jingzhu Shao^1,2^, Jiang Zhu^1,2^, Xiangyu Zhao^1,2^ and Chongzhao Wu^*1,2^

^1^ Center for Biophotonics, Institute of Medical Robotics, Shanghai Jiao Tong University, Shanghai, China

^2^ School of Biomedical Engineering, Shanghai Jiao Tong University, Shanghai, China

^‡^ These authors contributed equally to this work.

^*^ Corresponding author: [*czwu@sjtu.edu.cn*](mailto:czwu@sjtu.edu.cn)

# Ⅰ. Influence of neural-network parameters on the performance of the proposed method

Many critical parameters in the neural network module would influence the performance of the proposed method, such as different numbers of iterations and different normalizations for target electric field distribution, and their corresponding influences have been illustrated in Figure 5. Apart from that, some other parameters influence the reconstruction quality as well, including loss functions, batch size, the amount of training samples, and even the internal architecture of our adopted neural network.

Firstly, the influences of different loss functions are analyzed here. Both MSE and NPCC have demonstrated their high effectiveness and excellence in the field of error estimation during the past several decades. However, for the case in this work, only MSE or only NPCC is not powerful enough to serve as the loss function. Figure S1 shows the reconstruction comparison employing different loss functions, i.e., MSE loss, NPCC loss, and the combined loss. When only MSE loss is adopted, the reconstruction can obtain a clean background but suffers from discontinuous edges within the target region. In contrast, when only NPCC loss is adopted, the reconstruction keeps a better shape in target region but suffers from noisy background. Therefore, we combine these two loss functions for a better reconstruction with both continuous edges and clean background. Moreover, from a quantitative point of view, a higher peak signal-to-noise ratio (PSNR) of the combined loss also demonstrates its superiority over a single MSE or NPCC loss.

Furthermore, as shown in Figure S2, the influence of other parameters, such as batch size (*N*_batch_), amount of training data (*N*_data_) and number of residual blocks (*N*_block_) inside the proposed neural network, has been studied as well. A larger batch size and larger amount of training data will lead to a more precise target light modulation with a higher PSNR. In terms of numbers of residual blocks inside neural network, 5 residual blocks achieve a higher PSNR than 2,3 and 4 residual blocks in neural networks. It can also be found that compared with GS algorithm, different values of a single parameter in the neural network have relatively little impact on the performance of our model. Even the lowest PSNR among them (PSNR = 15.49 when *N*_data_=100) is still higher than that of GS algorithm (PSNR = 13.12 as shown in Figure S2(b)).


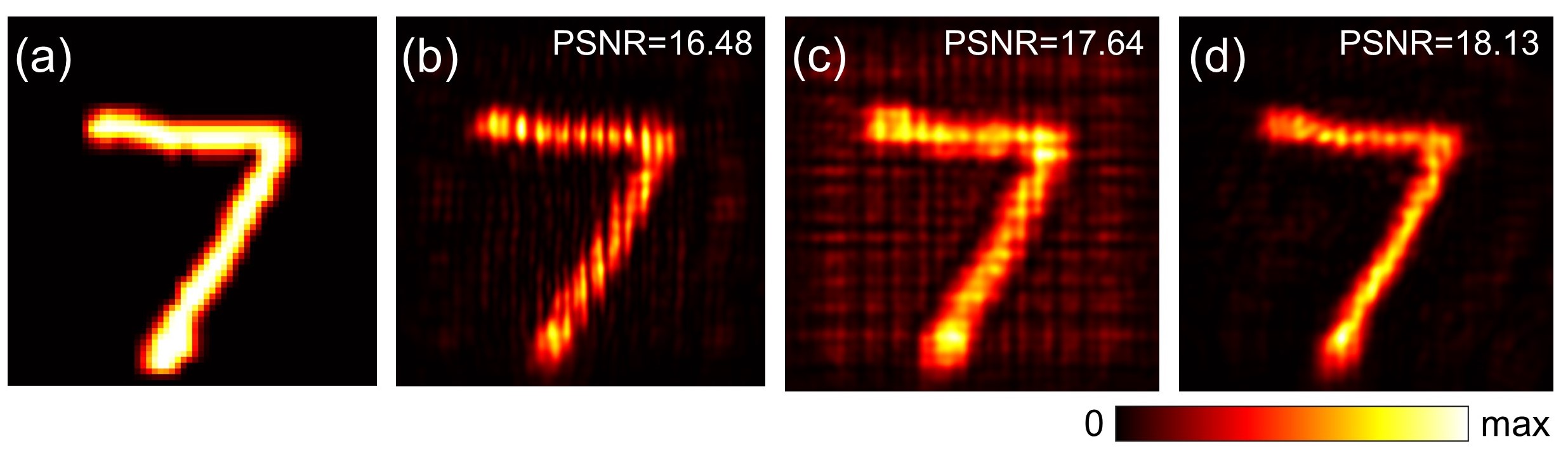


**Figure S1:** Intensity distribution of reconstructive images when utilizing loss functions. (a) Target image. (b) Only MSE as the loss function. (c) Only NPCC as the loss function. (d) Combination of MSE and NPCC as the loss function.


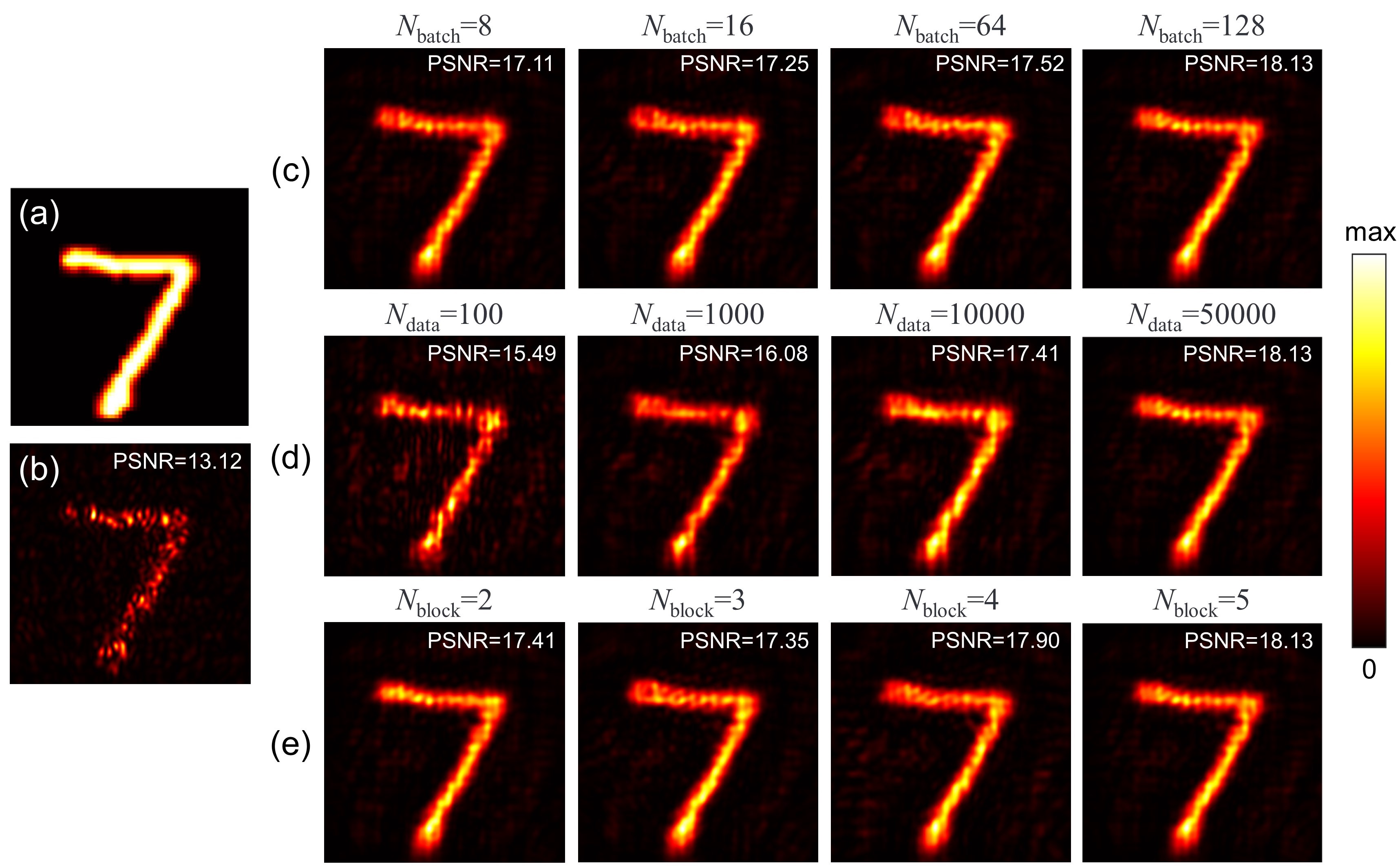


**Figure S2:** Comparison of performance with different parameters of the proposed model. (a) The target electric field distribution. (b) Result from GS algorithm. (c)-(e) Results from different batch sizes (*N*_batch_), amounts of training data (*N*_data_) and numbers of residual blocks inside Y-Net (*N*_block_), respectively.

# Ⅱ. Performance of the proposed method about different array dimensions of holograms

The scalability of the proposed method has been studied by end-to-end inverse design of larger-scale metasurface holograms with array dimensions of 128 × 128, 256 × 256, 512 × 512, 1024 × 1024, respectively. In the cases of different array dimensions above, the training procedure takes a longer time as the designing array becomes larger, varying from around 3 hours for 128 × 128 array, up to around 6 hours for 1024 × 1024 array. The time to predict the parameter array with different array dimensions has been recorded and compared with GS algorithm as shown in Table S1. It can be observed that with the increase of array dimensions, running time of GS algorithm for a single data sample increases dramatically from 0.36 s to 113.35 s. However, our proposed method remains highly efficient, and running time is consistently within several seconds, even for the array dimensions up to 1024 × 1024.

Considering that the required memory and computational complexity for simulations with cylinder array larger than 64 × 64 are beyond the best performance of our computer hardware, we utilize angular spectrum propagation method to validate the performance of the proposed method with different array dimensions. Figure S3 shows the reconstruction results with different array dimensions, and we can clearly see that all the background of reconstruction is clean and all the energy distribution of electric field is confined in the target region with a sharp outline, suggesting the proposed method will be potentially applied to large-scale holographic displays with subwavelength resolution.

**Table S1:** Comparison of running time between Physics-driven Deep Neural Network (PDNN) and the Gerchberg-Saxton (GS) algorithm for a single data sample with different array dimensions.

| Array dimension | 64×64 | 128×128 | 256×256 | 512×512 | 1024×1024 |
| --- | --- | --- | --- | --- | --- |
| Running time (PDNN) | 0.47 s | 0.52 s | 0.68 s | 1.24 s | 3.21 s |
| Running time (GS) | 0.36 s | 1.34 s | 6.55 s | 26.91 s | 113.35 s |


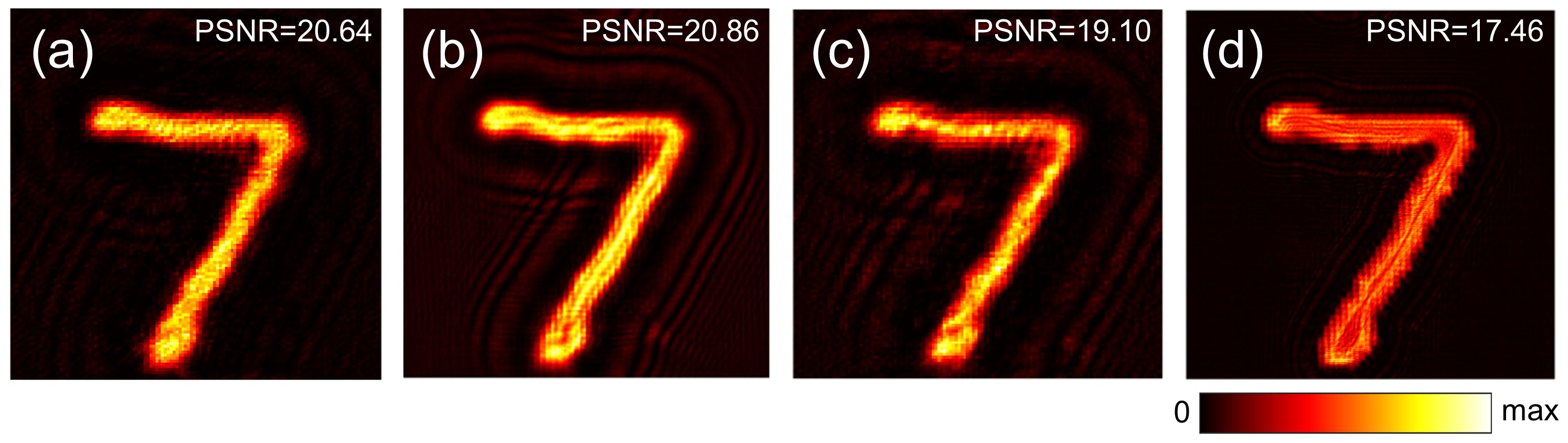


**Figure S3:** Intensity distribution of reconstructive images when different array dimensions are adopted. (a) 128×128 array. (b) 256 × 256 array. (c) 512 × 512 array. (d) 1024 × 1024 array.
